# Tinnitus and Other Auditory Problems – Occupational Noise Exposure below Risk Limits May Cause Inner Ear Dysfunction

**DOI:** 10.1371/journal.pone.0097377

**Published:** 2014-05-14

**Authors:** Ann-Cathrine Lindblad, Ulf Rosenhall, Åke Olofsson, Björn Hagerman

**Affiliations:** 1 Department of Clinical Science, Intervention and Technology, Division of Ear, Nose and Throat Diseases, Unit of Technical and Experimental Audiology, Karolinska Institutet, Stockholm, Sweden; 2 Department of Clinical Science, Intervention and Technology, Division of Ear, Nose and Throat Diseases, Karolinska Institutet; and Department of Audiology and Neurotology, Karolinska University Hospital, Stockholm, Sweden; University of Salamanca- Institute for Neuroscience of Castille and Leon and Medical School, Spain

## Abstract

The aim of the investigation was to study if dysfunctions associated to the cochlea or its regulatory system can be found, and possibly explain hearing problems in subjects with normal or near-normal audiograms. The design was a prospective study of subjects recruited from the general population. The included subjects were persons with auditory problems who had normal, or near-normal, pure tone hearing thresholds, who could be included in one of three subgroups: teachers, *Education*; people working with music, *Music*; and people with moderate or negligible noise exposure, *Other*. A fourth group included people with poorer pure tone hearing thresholds and a history of severe occupational noise, *Industry*. N_total_ = 193. The following hearing tests were used:

− pure tone audiometry with Békésy technique,

− transient evoked otoacoustic emissions and distortion product otoacoustic emissions, without and with contralateral noise;

− psychoacoustical modulation transfer function,

− forward masking,

− speech recognition in noise,

− tinnitus matching.

A questionnaire about occupations, noise exposure, stress/anxiety, muscular problems, medication, and heredity, was addressed to the participants. Forward masking results were significantly worse for *Education* and *Industry* than for the other groups, possibly associated to the inner hair cell area. Forward masking results were significantly correlated to louder matched tinnitus. For many subjects speech recognition in noise, left ear, did not increase in a normal way when the listening level was increased. Subjects hypersensitive to loud sound had significantly better speech recognition in noise at the lower test level than subjects not hypersensitive. Self-reported stress/anxiety was similar for all groups. In conclusion, hearing dysfunctions were found in subjects with tinnitus and other auditory problems, combined with normal or near-normal pure tone thresholds. The teachers, mostly regarded as a group exposed to noise below risk levels, had dysfunctions almost identical to those of the more exposed *Industry* group.

## Introduction

Hearing problems are not just a matter of reduced ability to recognize speech and other sounds, but also of tinnitus, abnormal sensitivity to loud sound and sound distortion. Hearing loss, as measured by pure tone audiometry, is often accompanied by tinnitus. However, tinnitus occurs without concurrent self-reported hearing loss in about 1/3rd of all cases[1] (but bear in mind that self-reported hearing loss is poorly correlated to audiometric status). There are some reasons for this: 1) Tinnitus can be a non-auditory symptom, not involving the peripheral auditory system[2]; 2) Tinnitus can occur in conjuncture with a subclinical dysfunction of the cochlea[3], [4], e.g. as a symptom at an early stage of noise-induced hearing loss (NIHL).

In a study of risk factors for tinnitus in a population 55 years and older Sindhusake et al[5] described a number of extrinsic and health factors that could be linked to tinnitus. The factor with the largest attributable risk was self-reported work-related noise exposure (almost 14%). The largest single factor was self-reported tolerable occupational noise exposure (9.3%). This means that a number of occupations with less noise burden than industrial exposure are at risk for noise-induced tinnitus. Teachers/preschool teachers are exposed to sudden slamming of for example lids, doors or toys, and preschool teachers may also suffer sudden screams near to the ear, although the mean noise levels are not excessive[6], [7]. In occupations related to music the noise often varies around the sound level above which there is a risk of NIHL according to the international standardisation[8]. However, music sounds can be very loud, and alterations in the audiogram indicative of NIHL have been reported[9], [10]. In other studies no relationship between music exposure and hearing loss, in terms of pure tone thresholds, has been found (for a review, see Zhao et al[11]). Tinnitus is common among rock/jazz musicians, also when the audiogram is similar to that of a reference group not exposed to excessive music[12].

These days when tinnitus is being paid so much attention, it may not be provocative to claim that hearing problems can be more than, or different from, having bad pure tone thresholds. Therefore there should be a demand for diagnostic test methods for identifying or excluding dysfunctions associated to the cochlea as the origin(s) of the hearing problems. Results from such tests could form the basis for individual treatment plans.

A test protocol, intended to be sufficiently sensitive to detect dysfunctions associated to minute cochlear lesions that cannot be diagnosed by routine clinical audiological tests, was developed. The protocol now consists of six auditory physiological tests, a Békésy audiogram, and a questionnaire.

In a first study, with fewer tests and test subjects (n = 46), who had tinnitus and normal or near-normal hearing-thresholds, we found that certain results could be associated with certain backgrounds[4]: 1) Persons exposed to impulse noise or other sudden loud sounds showed characteristic results. 2) Irregularities in the regulatory system may be a trustworthy sign of the cause of the tinnitus in persons well below middle-age, and with suspected hereditary tinnitus. 3) Subjects working with music had a variety of dysfunctions, but no features making them stand out from persons with other backgrounds. 4) Persons with suspected non-auditory tinnitus, on the other hand, showed very few dysfunctions.

The aim of the current study was to use the full test protocol to possibly identify dysfunctions associated with cochlear lesions in persons with up to moderate noise levels at work, with hearing problems, and without apparent deteriorations in the audiogram. If we found dysfunctions, would professions with different types of noise show their own characteristics?

The main finding of the current study was that teachers had characteristic results for two measurements not used in the first study. Those results were similar to results from industry workers tested for comparison, although the teachers had about 20 dB better pure tone thresholds. However, there were still no characteristic features to be found for musicians.

## Materials and Methods

### Ethics statement

The investigation was approved by the Regional ethical review board in Stockholm, no. 04-228/4. All test subjects gave their informed consents (written) to participate in the study.

### Test subjects

Altogether 272 subjects of working age with tinnitus or other hearing problems, and not wearing a hearing aid, were tested in the study. About half of them were recruited by announcements in hearing clinics in Stockholm. The other half was recruited by an announcement at the home page of Karolinska University Hospital, and participants came from all over the country. The subjects were recruited and measured during a period of three years. There was no medical examination of the test subjects. The characterization of symptoms and exposure to e.g. noise was entirely based on the questionnaire. As it happened, very few professions were represented among these volunteers. Thus groups were formed to fit the assortment of volunteers regarding the amount and characteristics of noise exposure. It was not possible to match the participants regarding gender and age. Therefore the results should be interpreted with caution. However, some of the results have been compared with results for groups of persons of similar ages, but without hearing problems, or with internationally standardised pure tone thresholds. Reported here are results of subjects, who fulfilled the threshold criterion of having normal or near-normal hearing thresholds and who could be included in one of three groups of professions. Results of a fourth group with industrial noise and worse hearing thresholds are also reported for comparison. This study may have found dysfunctions that are characteristic for these groups before the audiogram is affected, but it will not give us a general overview of the effects of noise on hearing for these groups of professions at later stages of deterioration of the audiogram. Neither can it tell how common the detected dysfunctions are.

The groups were: 1) teachers, with 1/3rd of them preschool teachers, forming the group *Education*; 2) people working professionally with music, the group *Music*; and 3) subjects with low or, in a few cases, moderate occupational noise exposure, mostly medical staff and students, the group *Other*. The group *Other* seemed the least homogenous regarding noise exposure. In these groups (1–3) pure tone thresholds on both ears had to be ≤25 dB hearing level (HL) at the frequencies 500, 1000, 1500 and 2000 Hz, and ≤40 dB HL at 4000 Hz. The average threshold at the frequencies 3000, 4000, and 6000 Hz on both ears had to be ≤35 dB HL. The threshold fence defining normality or near-normality was chosen to include the effect of normal aging for all the test subjects. At the higher frequencies it corresponds to the median thresholds of otologically normal 66 year old men with no history of undue noise exposure[13].

For comparison a fourth group was selected 4) constituted of persons with blue-collar occupations, having audiograms with high- (and sometimes mid-) frequency hearing impairments, indicative of noise-induced hearing loss, and also having tinnitus, the group *Industry*.

**Figure 1:**
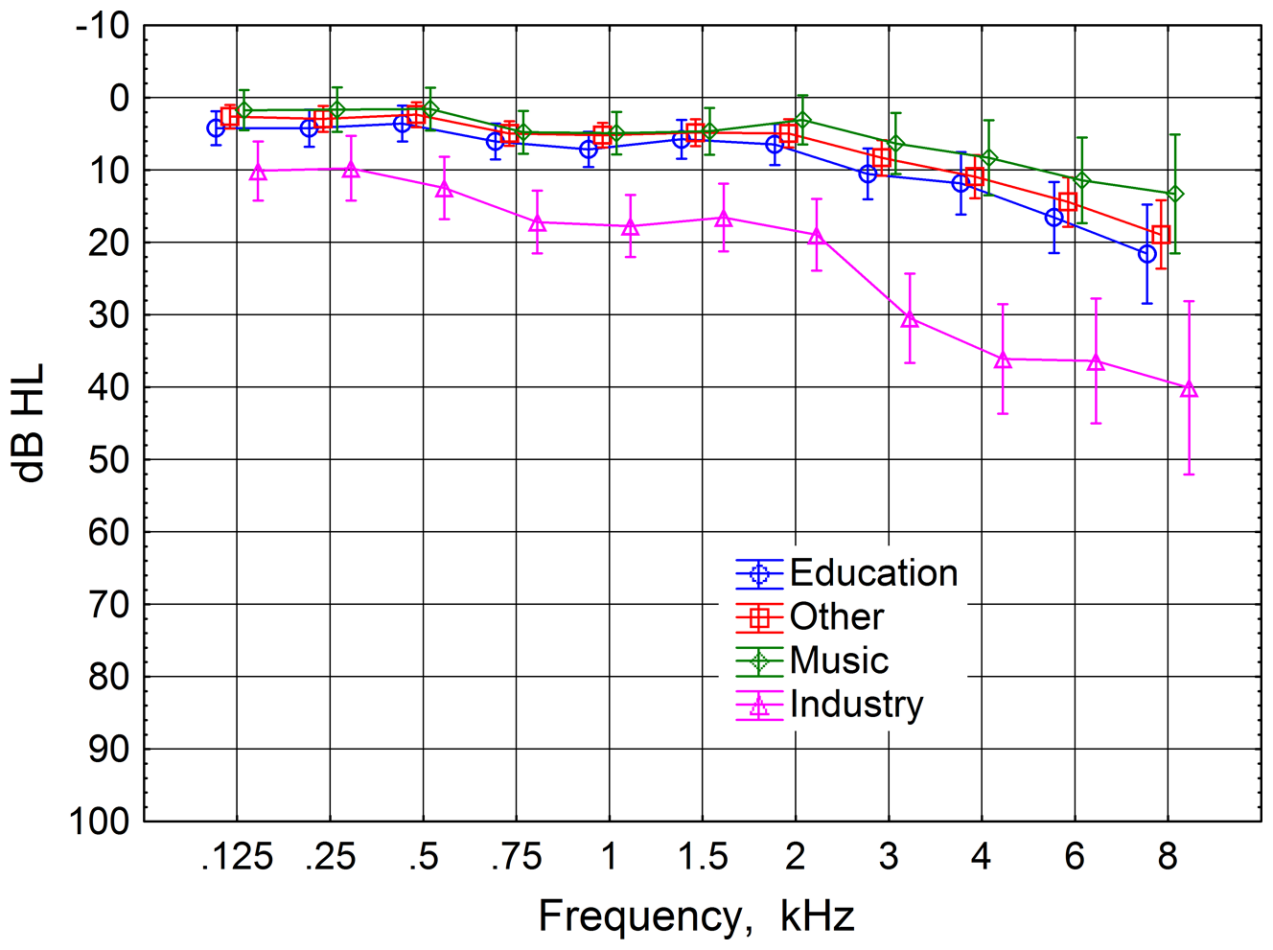
Mean audiograms for the profession groups. Group names as shown in Table 1. Please note that the group *Industry* had no threshold restrictions. The vertical bars represent 95% confidence intervals. The data points are horizontally displaced for clarity.

After omission of data from people not conforming to the threshold criterion or being unsuitable for the chosen groups of professions there was a total of 193 subjects, 102 men and 91 women, 18 to 66 years of age, to be analysed. The groups are presented in Table 1. The proportion of genders in the profession groups seems roughly the same as in working life in our country. Mean audiograms for the four test groups are shown in Figure 1.

**Table 1:** Number, age, and gender of subjects in groups.

Profession	Group name	Number	Mean age (SD)	No. of men	No. of women
Teachers	*Education*	48	45.4 (10.6)	9	39
Musicians	*Music*	32	37.8 (11.5)	20	12
Other	*Other*	97	39.2 (13.5)	57	40
Noisy industry	*Industry*	16	54.4 (9.3)	16	0
TOTAL		193	45.0 (13.0)	102	91

### Reference groups

“Normal” reference results for our methods and our equipment were used for comparison. These results were meant to be to be used in several projects. A *middle-aged reference group* consisted of fifty-seven 41–60 year old subjects, all without hearing problems and with hearing thresholds no worse than the median for 50-year-old men[13]. (In the 5-year age intervals there were 8 women and 8 men who were 41 to 45 years old, 7 women and 7 men who were 46 to 50, 7 women and 9 men who were 51 to 55, and 8 women and 4 men who were 56 to 60.) Another group consisted of twenty-seven young women, 18–24 years old, considering themselves normal and unexposed, and with normal hearing thresholds (<20 dB HL 125–8000 Hz except for four individual thresholds exceeding 20 but not 23 dB HL). The mean pure tone thresholds for the group turned out to be 5 dB HL. A comparison to the ISO-standard above tells that 80% of unexposed women at those ages have better thresholds than our group of young women. Several of these young women often went to discos or pop concerts and had tinnitus. Thus they were not an ideal, “normal”, “unexposed” group.

### Equipment and measurements

A computer-controlled Tucker-Davis Technologies (System III) module system was used with Sennheiser HDA 200 headphones at the psychoacoustical tests, and with amplifiers plus probe systems ER-10C from Etymotic Research on both ears for the measurements of otoacoustic emissions. The HDA 200 headphones were calibrated according to ISO 389-8 and the ER-10C probe systems were calibrated in an ear simulator according to IEC 60711.

The software for all the methods, for the computer and the Tucker-Davis equipment, was written in our laboratory. The TDT system was programmed to produce and present stimuli, for example the OAE stimuli and the contralateral noise, as well as collect and store the responses. However, the speech test used stored sound files for the words and their respective noise stimuli. The adaptive procedure of the speech test was controlled by the computer. All tests were performed in a sound proof audiometric test room.

Basic otoscopy and tympanometry were performed. When necessary, cerumen was removed.

The hearing measurements took about three and a half hours including a coffee break. All the participating subjects performed all the tests. However, individual test subjects were allowed to exclude stimulus levels they considered too high.

#### Questionnaire

In the intermission between the measurements the subjects were given a questionnaire. Apart from questions about age and profession there were 22 questions with two to eight response alternatives, sometimes also with a possibility of an open response. The questions regarded hearing problems, tinnitus, sensitivity to loud sounds, noise exposure at work and in their leisure time, musical activities, impulse noise incidents, military service, medication (pain relieving and tinnitus medicine), eye and hair colour, smoking, neck problems, headache, stress, and relatives with hearing problems. The questions on tinnitus and sensitivity to loud sounds had the response alternatives: never, only after a loud sound, at certain occasions, often, and constantly. The questions on headache and neck problems had only the response alternatives yes and no. The question “Do you feel stressed/anxious?” had the five response alternatives: never, seldom, sometimes, often and always. A translation of the questionnaire, including number of responses to each alternative, is presented in Appendix S1.

From the responses to the questions about profession, different types of noise exposure and exposure times, figures of noise exposure were estimated. The underlying principles for the judgements are found in Appendix S2. A summary is presented here: The range for 1) work noise exposure was 0 to 4, for 2) leisure time 0 to 3, and for 3) military service 0 to 2. Persons with only marginal noise exposure in compulsory military service, being well protected during a few initial shooting exercises, got the estimate 0, the same as persons not having been in military service. The total noise exposure was calculated as the sum of the three figures, which gave a scale from 0 to 9. However, the highest value for an individual in this study was 4. Within each profession group the figures were fairly equal. 4) Self-reported incidents with impulse noise were treated separately, range 0 to 3.

#### Békésy audiometry

Pure tone thresholds for left and right ears separately were measured at 125, 250, 500, 750, 1000, 1500, 2000, 3000, 4000, 6000, and 8000 Hz.

A pulsating tone is presented. The duration is 225 ms, including attack and release times, and with a 175 ms long interval between pulses. The level of the tone is increased by 2.8 dB/s until the subject detects the tone and starts pressing the button. The level of the tone decreases with the same speed until the button is released again, etc. Thus a zigzag pattern is formed around the threshold level. The turning-points are registered by the computer. The measurement is concluded after 10 turning points. The first two turning points are not used in the calculation. The threshold is calculated as the mean value of the medians of the remaining upper and lower turning points, and presented with the resolution of 1 dB. These parameters should give high accuracy with a standard deviation of repeated measures not exceeding 1.8 dB[14].

#### Otoacoustic emissions, TEOAEs

Transient evoked otoacoustic emissions, TEOAEs, were measured at two input levels without or with contralateral noise. Normally the contralateral noise will make the efferent system controlling the outer hair cells, OHCs, reduce, suppress, the response amplitude. We measure the response correlated to the stimulus signal, but also the uncorrelated response.

Clicks with the duration of 80 µs were repeated with a frequency of 50 Hz. The measurement was performed in a nonlinear mode to enhance those components in the response, which have a nonlinear dependence of the stimulus level, and to suppress the linear components. To accomplish this, the polarity of every fourth click is reversed and the sound pressure level is increased by a factor of 3. After removal of the primary click by windowing technique, the acoustical responses from 1000 clicks are averaged. The stimulus level is specified as so called peak equivalent sound pressure level, peSPL. TEOAEs were measured at 70 and 85 dB peSPL with and without contralateral masking consisting of a 70 dB SPL broadband noise. The RMS-values for the correlated and uncorrelated responses, over the interval of measurement, are used as variables in the analyses and so are the RMS-values in three different frequency areas, Low (500–2500 Hz), Mid (2500–5000 Hz), and High (5000–8000 Hz).

#### Otoacoustic emissions, DPOAEs

When measuring distortion product otoacoustic emissions, DPOAEs, two stimulus tones are given, with frequencies near each other, f_2_/f_1_ = 1.25, and near the measurement frequency. The measurements were performed on both ears at an input level, L, of f_1_ of 65 dB SPL, L_f1_/L_f2_ = 10 dB, nominal frequency √f_1_*√f_2_. A weak response is measured at the frequency 2f_1_–f_2_. Two measurements were done, the first one at the nominal frequency 1000 Hz and the second one at 2000 Hz. To avoid measuring in a notch of the DPOAE microstructure an automatic procedure measured at a few frequencies between 0.95 and 1.05 times the nominal frequency with an increment of 0.01 times the nominal frequency. The measurements were then performed at the frequency with the largest response. To estimate the background noise from equipment and ear, the level in narrow frequency areas below and above the response tone frequency was measured.

The measurements were made without and with contralateral noise. We made 20 3s long measurements of the response from the two tones without contralateral noise. We calculated the individual standard deviation of these responses to test the stability.

Measurements were also made with on-off noise (12 s on, and 12 s off, 5 times) on the contralateral ear in order to investigate the ability of the ear to regulate the sensitivity of the other ear. The response normally decreases somewhat when the contralateral noise is on. The difference between the medians of the five off-periods and the medians of the five on-periods shows the ability and endurance of the ear to suppress the response when the noise is on.

#### Psychoacoustical modulation transfer function, PMTF

The active nonlinear process in the cochlea is mediated by the OHCs and facilitates the perception of the complex sound patterns in speech. These patterns are characterized by rapid sound variations combined with slow modulations caused by speech syllables, words and intonation. A measurement termed the psychoacoustical modulation transfer function (PMTF) reflects the functioning of the inner ear when handling slow intensity variations like those of speech. PMTF measures the thresholds of brief tones placed at the peaks or in the valleys of a fully, sinusoidally intensity-modulated, octave-band noise at various sound pressure levels centred at the test tone frequency[15], thus involving simultaneous, forward and backward masking. The measurements were performed with Békésy-technique at various sound pressure levels of the noise. The test has been shown to measure more subtle qualities of hearing, and there is evidence that it reflects hair cell function[15].

*PMTF measurements*: The measurements were performed with the brief tone, 4 ms with raised cosine flanks, first with the octave-band filtered noise at 2000 Hz, and with a modulation frequency of 2.5 Hz; and then with the octave-band filtered noise at 4000 Hz, and with a modulation frequency of 10 Hz. The noise levels were 35 to 85 dB SPL in steps of 10 dB. Initially, the threshold for the brief tone was measured without noise to familiarize the subject with the brief tone. Both ears were measured, with the left ear first. When the subject expressed tiredness or discomfort, only one ear, mostly the left ear, was measured.

**Figure 2:**
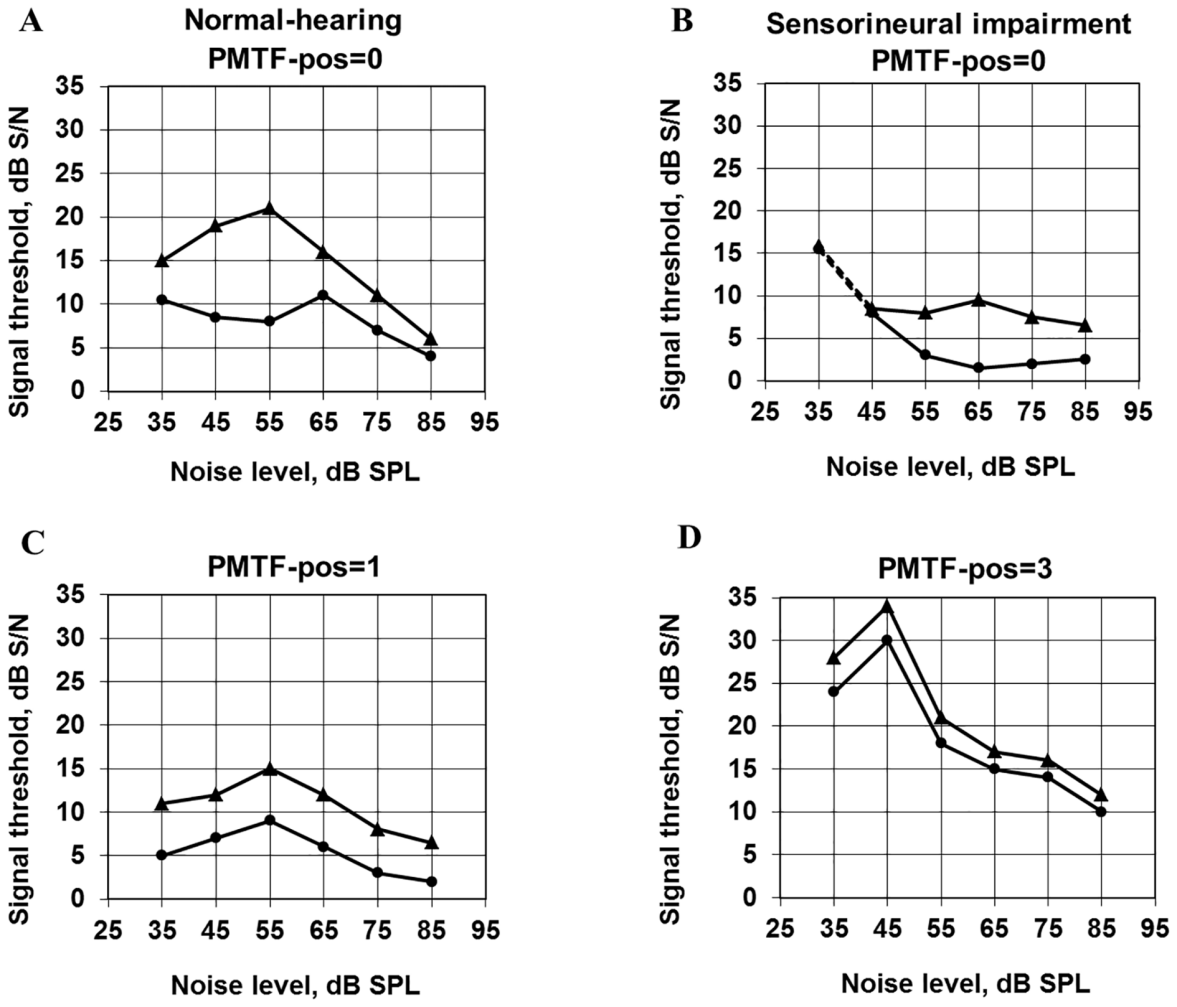
Characteristic PMTF-curves. The PMTF-curves are classified as four different P-types. 0 = normal PMTF-curves (2A) or typical sensorineural loss (2B) that is still measurable; 1 = mildly abnormal (2C), see text; 2 = suspected hyper-PMTF (not shown in the figure); 3 = hyper-PMTF (2D), indicates damage from impulse noise or other sudden, loud noise with a rapid onset; NM = not measurable (not shown in the figure). Triangles indicate peak thresholds. Round markers indicate valley thresholds.

*Typical PMTF results*: Figure 2 shows a few stylized, but characteristic PMTF curves. The normal PMTF curves are nonlinear, with a maximum in signal-to-noise-ratio, S/N, for the peak threshold, occurring at a noise level around 55 dB SPL (Figure 2A). (The term *peak threshold* is used for the threshold of the brief tone when the tone is placed at the peak of the noise. The term *valley threshold* is used when the tone is placed in the valley of the noise.) For the valley threshold there is a corresponding maximum at about 65 dB SPL (Figure 2A).

For a sensorineural hearing loss of cochlear origin, the nonlinearity is weaker. Both maxima have lower S/N and they occur at higher noise levels (Figure 2B). This type of pattern indicates *reduced nonlinearity*. However, in this study we focused on the effects described in the following paragraphs.

A second type of abnormal PMTF pattern is presented in (Figure 2D). The S/N maxima of the peak and valley threshold curves have markedly increased amplitudes and occur at the same, low noise level (35–45 dB SPL). The peak and valley curves are almost identical, which implicates that the affected ear can hardly take advantage of the silent interval around the brief tone in the valley. The term “hyper-PMTF” was coined for this pattern, which can appear after unprotected exposure to impulse noise or other sudden, loud noise with a rapid onset[4]. We speculate that there are lesions in the inner hair cell, IHC, region, since impulse noise by itself or embedded in continuous noise causes a larger proportion of damage to IHCs than continuous noise[16].

There are also intermediate varieties between normal and hyper-PMTF. A mildly abnormal variety is shown in Figure 2C. Like in the hyper-PMTF the S/N maxima of the peak and valley curves are positioned at the same noise level, but here they occur at a normal noise level (>45 dB SPL), and they are not as high as in the hyper-PMTF. The level dependence for peak and valley curves is the same. At every noise level used the S/N for the peak threshold is a roughly constant number of dB higher than the S/N for the valley threshold.

It is evident from the examples above that there are two main types of abnormal PMTF results: The positions of the maximum peak and valley thresholds can occur at higher (Figure 2B) or lower (Figure 2D) than normal noise levels (horizontal position in graph), and the corresponding S/N maxima are then either lower (Figure 2B) or higher (Figure 2D) than normal (vertical position in graph). Therefore, a practical way of analysis is to use a qualitative method to define certain typical patterns, for example the hyper-PMTF.

Depending on combinations of shapes and positioning of the peak and valley curves (relative each other and regarding the noise levels of the maxima) we divided the PMTF curves into classes depending on the likeness to hyper-PMTF. We called them P-type, and numbered them 0 to 3. A few P-types are exemplified in Figure 2. Curves denoted P-type = 3, called hyper-PMTF, indicate dysfunction, possibly from impulse noise or other sudden, loud noise with a rapid onset, Figure 2D. P-type = 0 means normal, or typical sensorineural loss that is still measurable at fairly low noise levels, Figure 2A and 2B. P-type  = 1, seen in Figure 2C, and P-type = 2 fall in between: P-type =  1 looks more like P-type = 0; and P-type = 2 looks more like P-type = 3 by showing curves with maxima at low noise levels but not as high and/or regular as in the typical hyper-PMTF, i.e. “suspected hyper-PMTF”. P-type = NM means not measurable, i.e. the peak and valley thresholds for the brief tone are entirely determined by the threshold for the brief tone without noise at the low noise levels where the characteristics of the hyper-PMTF are seen.

#### Forward masking

Experiments on mice, using auditory brainstem responses[17], have suggested that forward masking results indicate the status of the IHCs. After the offset of a noise burst the masking effect on a brief tone decreases rapidly in a good ear, but, according to that article, not in an ear with loss of IHCs. In our project a brief tone, 4 ms, at 2000 Hz (the same as used in the PMTF measurements), is presented 5 or 50 ms after the offset of an octave-band noise burst of 400 ms duration and a repetition interval of 800 ms at 65, 75 or 85 dB SPL, and the corresponding thresholds are measured with Békésy technique for one ear at a time, both ears. The difference between the thresholds for 5 and 50 ms interval between noise burst and tone at the same noise level is calculated. A small difference means that the masking effect from the noise burst has not decreased much after 50 ms, which has been attributed to less well-functioning IHCs[17], [18]. Our middle-aged reference group showed a threshold difference of 27 ±9 dB. The median was also 27 dB, with lower quartile 22 dB, and a maximum of 49 dB. With results at the lower quartile the speech recognition in noise seemed to start to be degraded. For the group of young women the threshold difference was just 1 dB larger, 28 ±11dB, but the median was 30 dB, and the maximum threshold difference 51 dB.

#### Speech recognition threshold in modulated noise

The speech recognition threshold in modulated noise was measured on the self-reported worst ear. Hagermańs 5-word sentences in noise were used with the original versions of the various words stored in the computer[19]. The noise was modulated to a degree of 100%, i.e. as much as possible without getting overflow[20], and had a long-term average spectrum identical to that of the speech read by a female voice. The modulating signal was a noise with most of its energy between 1 and 5 Hz, and a spectrum similar to the modulation spectrum of normal speech[19]. An adaptive method was used for the threshold measurement, with the change of the speech level after each sentence depending on number of correct words obtained. For a more detailed description see [20]. The threshold is defined as S/N for 40% correct words. The measurements were performed at noise levels 70 and 85 dB SPL. When 85 dB SPL was considered too loud, both ears were measured at 70 dB SPL. For the *middle-aged reference group* the S/N at threshold, over both ears, were −13.6 ±2.1 dB, at 70 dB SPL presentation level, and −15.5 ±2.2 dB, at 85 dB SPL. Corresponding values for the *group of young women*, for the left ear only, were −15.4 ±0.9 and −16.3 ±1.1 dB.

#### Tinnitus matching

The subjects were asked to compare pure tones from a modified version of the Békésy audiometry program to match the pitch of their tinnitus. The level was changed in steps of 1 dB, and the frequency in steps of 1 Hz. The participants were asked to: “Try to match the frequency of the tone in the earphone with the pitch of your tinnitus. After that, match the level of the tone to that of your tinnitus”. The drawback with this method is that it is only applicable to tinnitus with a tonal character. The matching is less accurate for those perceiving their tinnitus as noise or other non-tonal sounds.

#### Statistical analyses

The program Statistica 9.1 was used for statistical analyses. For the hearing measurements, all of them resulting in continuous variables, analysis of variances was used, often with subsequent Tukey's post hoc tests. To find relations between continuous variables linear regression with age as covariate was used. However, in analyses where age was not significant, results from ANOVAS without age as covariate are presented. For the variables from the questionnaire, with rank order variables, where no information is given in the text, Kruskal-Wallis ANOVA by ranks was used, sometimes with subsequent multiple comparisons. Other statistical tests are mentioned in Results and Discussion.

## Results and Discussion

### Questionnaire

#### Hearing problems

Among the 193 subjects analysed, 69% had tinnitus and were also hypersensitive to loud sound, 22% had tinnitus only, 7% were only hypersensitive to loud sound and 2% had neither of these two symptoms, but felt they had difficulties hearing in noise. On a general question about hearing problems 25% answered that their hearing symptoms disturb them always: affect sleep, affect their whole life; 28% that their symptoms affect them in normal sound environments, but their tinnitus is masked by louder sounds; 25% that their symptoms affect them only in a quiet environment; 7% that they have symptoms but no problems (although the criterion for participating was having problems); 13% said they have problems, but did not specify to what extent. There was no statistical difference between the profession groups in this respect. Neither was there any difference between groups regarding hypersensitivity to loud sound.

#### Noise exposure

Very rough estimates of noise exposure based on the individual responses to the questionnaire indicated that the groups *Education* and *Other* were significantly different from the groups *Music* and *Industry* by being judged less noise exposed (p<0.05). This was the result of a Kruskal-Wallis ANOVA by ranks with estimated noise exposure as the dependent variable and with profession group as the between subjects factor. The questions referred to regarded profession, number of years working, leisure activities, and military service.

Regarding self-reported incidents with impulse noise or sudden very loud noise, a corresponding analysis showed that the exposure estimate for the *Industry* group was significantly higher than for the three other groups (p<0.01). Another group, which might have reported incidents for example with positive feedback in electronic systems, is the *Music* group. In our study, however, the musicians claimed that they were not using electronic amplification. This should have decreased the risk of unexpected sudden changes of the sound level. And the number of reported incidents did not differ significantly from those of the groups *Education* and *Other*.

The *Other* group included mainly hospital staff, and students. Only few of the participants had been exposed to occupational noise. In those cases, they handled medical devices.

#### Other data from the questionnaire

Regarding neck problems, *Education* had significantly higher values than *Industry* (p<0.0005, Pearson Chi-square). The *Education* group had significantly more self-reported tension headaches than the groups *Other* and *Industry* (p<0.05, Pearson Chi-square). The groups *Education* and *Music* had similar, slightly higher values for stress/anxiety than *Industry* and *Other*, but the differences were not significant. There were no significant differences between groups regarding pain relieving medication, eye and hair colour, smoking, and relatives with hearing problems. Tinnitus medicine was only used by ten subjects. Therefore it was not subjected to analyses. There was no difference in stress problems between the subgroups preschool teachers and other teachers.

#### Some relations between data from the questionnaire

Self-reported stress seemed to give tension headache and neck problems (p<0.05, p<0.005; binomial logit model). Headaches and neck problems influenced the use of medication (p<0.0001, p<0.05, Kruskal-Wallis ANOVA by ranks). The amount of medication was not influenced by age (ordinal logit model), gender or stress.

### Békésy audiometry

An ANOVA was performed with hearing threshold as the dependent variable, with profession group as a between subjects factor, and with ear and frequency as within subjects factors. The group *Industry*, the blue-collar group without threshold restrictions, chosen to have worse noise exposure than the other groups, was significantly different from all the other groups for left and right ears combined (p<0.00001, Tukey's HSD), Figure 1. For the *Industry* group the right ear was on average 2.8 dB worse than the left ear for all frequencies, but the difference was not significant. This group had worse pure tone thresholds than those of unexposed men of the same age[13].

### TEOAES

An ANOVA with the correlated TEOAE response as the dependent variable, with profession group as between subjects factor; and with ear, stimulus level, and frequency area as within subjects independent factors; showed no significant difference between the various professions or between left and right ear. For normal ears the response increases when the stimulus level is increased from 75 to 85 dB peSPL. There was such increases of 4.4 dB for each of the groups *Education*, *Other* and *Music*, and they were highly significant (p<0.00005, Tukey's HSD). However, when compensated for age they were not, and the response of the *Industry* group at the higher level was just marginally higher, 1.4 dB, than at the lower level. For the uncorrelated response signal, on the other hand, the *Industry* group showed a 6 dB higher response than *Education*, *Other* and *Music* (p<0.005 for all the three comparisons, Tukey's HSD). The TEOAE results of the *Industry* group may be explained by dysfunctional OHCs with reduced ipsilateral restraining regulation.

The contralateral suppression effect was very small, the means for the groups ranging from 0.22 dB to −0.26 dB without significant differences, although age was a significant factor.

### DPOAES

An ANOVA with the DPOAE response level without contralateral noise as the dependent variable, with profession group as between subjects factor; with ear and frequency as within subjects factors, and age as covariate, showed that the mean response level was significantly higher at 1000 Hz than at 2000 Hz, 7.5 and 3.7 dB SPL respectively (p<0.00001). There was a strongly significant age dependence (p<0.00001). From the right ear *Industry* had a very weak mean response over frequencies, −1.9 dB SPL, which was significantly lower than the responses of the other groups (p<0.005, Tukey's HSD).

To estimate the background noise from equipment and ear, the level in narrow frequency areas below and above the response tone frequency was measured. A statistical analysis corresponding to the one above was performed, now with this noise level as the dependent variable. In the left ear this noise level was about 3 dB higher than in the right ear (p<0.00001). Furthermore, the group *Industry* had an almost 3 dB higher value of that noise in the left ear than the groups *Education*, *Other*, and *Music* (p<0.05, Tukey's HSD).

A corresponding statistical analysis was also performed with the individual standard deviation of 20 consecutive measurements as the dependent variable. Also for this standard deviation the group *Industry* had a significantly higher value, 4.0 dB, than the groups *Education*, *Other*, and *Music* (2.0–2.1 dB), when averaged over ears and frequencies (p<0.00005). Age had a significant influence, but compensation for age did not change any values. Thus, the group *Industry* differed from the other groups by low DPOAE responses on the right ear, a high noise level around the tone response on the left ear, and a large variation in the 20 measurements without contralateral noise. It looks like the DPOAE response from this group mostly consists of noise. Compare to TEOAE results.

There was no significant influence of any of the factors ear, frequency or profession group on the suppression effect using the contralateral on-off noise. The range of suppression for the groups was 0.4 to 0.6 dB.

#### OAEs in short

As the *Industry* group was chosen to have a noise induced hearing loss it was a natural finding that *both types of OAEs* for the *Industry* group were marginal, unstable and considerably noisy. There were deteriorations in the contralateral regulatory system for all groups, but considerably more so in the *Industry* group. A few individuals in the other three groups had abnormally high emissions at some frequencies suggesting impaired ipsilateral regulatory function.

As a whole our results regarding OAEs did not give much information, probably because most of our test subjects were middle-aged. One should keep in mind that there is a normal degradation of OAEs and regulatory efferent system with age, which may obscure for example noise induced degradation [21].

### PMTF

The stimulus noise levels at which the maximum values of the threshold curves were found, were used as dependent variables in a multivariate ANOVA with profession group as between subjects factor; and with peak/valley curve and frequency as within subjects factors. Those noise levels did not differ significantly between the groups. This was true for both peak and valley curves and both ears.

The corresponding analyses, with the maximum value of the PMTF threshold curve on the left ear as dependent variable, showed that the maximum of the valley curve was significantly higher for the *Education* group than for *Other* and *Music* (p<0.05, Tukey's HSD). The same tendency was found on the right ear, but without statistical significance. An analysis for different types of teachers showed a tendency to higher maxima for preschool teachers than for other teachers. In this context it should be noted that a high maximum, especially on the valley curve, is one characteristic of a hyper-PMTF, Figure 2D. Although one cannot be certain that the results of the *Education* group are caused by sudden loud sounds, they definitely indicate a reduced ability to detect short sounds in silent intervals of a complicated signal, poor temporal processing.

**Figure 3:**
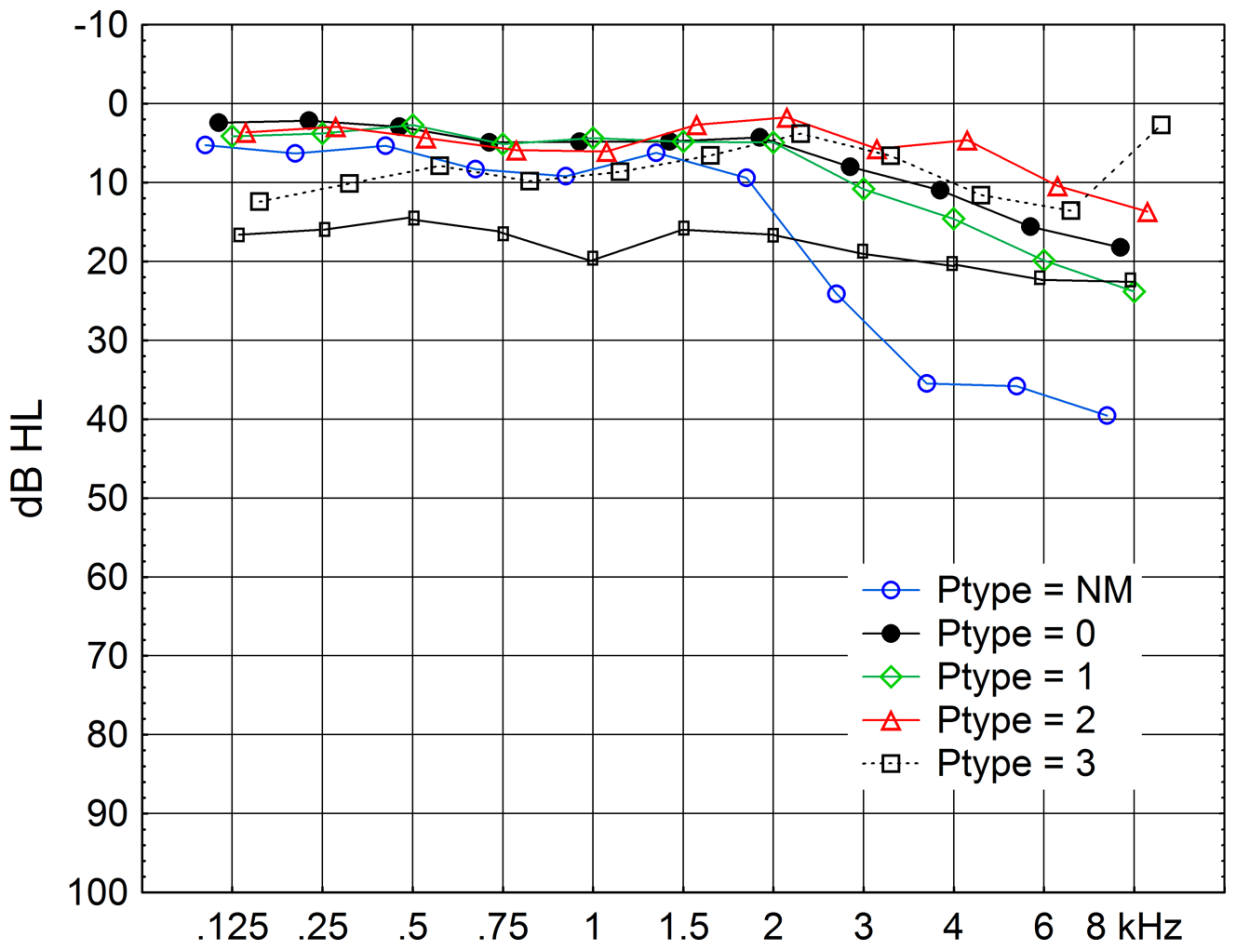
Mean audiograms for subjects with different types of PMTF-curves, 4000 Hz, left ear. See legend of Figure 2 about the PMTF classification. Numbers of subjects for P-types NM, 0, 1, 2, and 3 are 12, 40, 54, 13, and 2, respectively. An extra curve, black continuous line with smaller markers, black boxes, is added, which includes 4 more subjects with hyper-PMTF (P-type = 3), but outside the threshold criterion limiting to 193 subjects. Note that the two curves for hyper-PMTF are fairly flat. The data points are horizontally displaced for clarity.

Very few subjects with fully developed hyper-PMTFs (P-type = 3), suggesting exposure to impulse noise possibly associated to damage in the inner hair cell area [4], [16], [22], were found in this study. Although the *Industry* group had more self-reported impulse noise incidents (p<0.01) than the other groups, this did not result in significantly more hyper-PMTFs than in the other groups. One reason may be that some of the test subjects, especially in the *Industry* group, could not be measured at the low levels of the modulated noise at which the characteristics of the hyper-PMTF are seen. Measurements closer to the incidents, before the characteristics in our PMTF-measurements had been obscured by further deterioration of pure tone thresholds, would have clarified if there was a hyper-PMTF. Those actually classified with P-type = 3, hyper-PMTF showed fairly flat audiogram curves. See Figure 3. In the figure there is one curve showing the mean pure tone thresholds for the two subjects among the 193 who had P-type = 3 on the left ear. This curve shows thresholds around 10 dB HL. There is also an extra curve for these two subjects plus four more subjects with P-type = 3, left ear, from the total of 272 subjects measured. Most thresholds forming this curve are slightly better than 20 dB HL. Also the curve representing the 13 ears with P-type = 2, suspected hyper-PMTF, is fairly flat.

*Audiograms* with similar hearing thresholds for all frequencies have also been observed in conscripts sent to us for measurements after severe shooting incidents, unpublished data. It is not unusual that all thresholds have positions around 10 dB HL, i.e. totally normal. This may be a warning flag when a person with normal thresholds complains of hearing problems. In experiments on chinchilla, Wang[23] and El-Badry[24] have shown that as much as 70 – 85% of the IHCs could be damaged before threshold levels became abnormal. Another paper[25] shows that at sound levels well above threshold, other hearing functions, including forward masking, were strongly deteriorated before such a degree of degeneration.

### Forward masking

An ANOVA was performed with the forward masking threshold difference (difference between the thresholds of the brief tone at 5 ms and at 50 ms after the noise burst) as the dependent variable, with profession group as a between subjects factor, and with ear and level as within subjects factors. The two older groups *Industry*, mean age 54, and *Education*, mean age 45, showed significantly worse forward masking mean results (over ears and levels), 19 and 21 dB threshold difference, than the about five years younger groups *Music* and *Other*, 32 and 30 dB, (p<0.005, Tukey's HSD). These results could be compared to the threshold difference, 27 ±9 dB (median 27 dB, lower quartile 22 dB) for our *middle-aged reference group* without hearing problems. Most notable is that the median of the teachers, the *Education* group, 23 dB, was almost as low as the lower quartile of the *middle-aged reference group,* 22 dB. The lower quartile of *Education* was 12 dB.

On the other hand one may note that the results of *Music* and *Other* are as good as the median result of the (much younger) *group of young women*, 30 dB. Walton[26] found that aging changed forward masking for the worse. This was also the general effect over all our test subjects. The change was 3 dB per decade (p<0.00001 in all six linear regressions, i.e. two ears times three sound levels). However, in our study the significant differences between profession groups became even stronger when compensated for age (p<0.001, Tukey's HSD with age as covariate).

**Figure 4:**
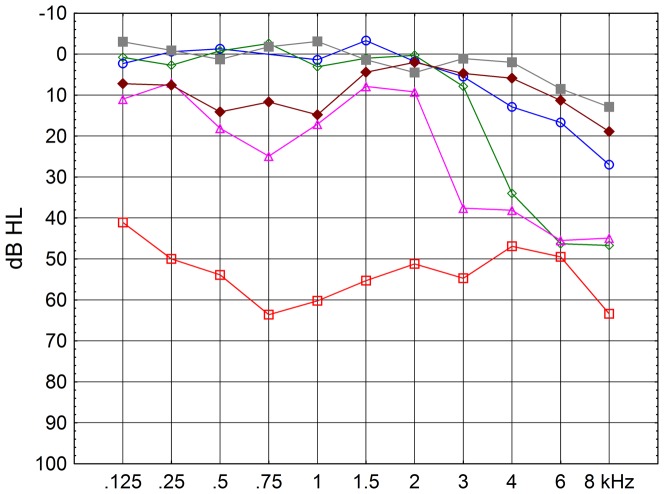
Individual audiograms for subjects with a forward masking threshold difference of less than 5 dB. Left ear, n = 6. The three worst audiograms come from the group *Industry* without restrictions on pure tone thresholds.

Figure 4 shows individual audiograms for six subjects with a threshold difference less than 5 dB for 5 and 50 ms delay between short tone and noise, on the left ear. Three of them came from the *Industry* group, two from *Education* and one from *Other.* Their PMTF results suggested either exposure to impulse noise or other sudden loud noise or they could not be measured at the levels of the modulated noise at which the characteristics of the hyper-PMTF are seen.

If an analogy with research animals is proposed, abnormal forward masking results may indicate IHC dysfunction or radial dendrite damage, independent of OHC status[18]. Later research on animals has found other damage in the IHC area [27]–[31], from earlier noise exposure not causing permanent threshold shifts, but with supra-threshold effects. If the speculation is true, that the ears of the research animals and humans work in a similar way, the results of this study suggest that some persons with professions considered less noise exposed may have considerable damage in the IHC area – and more such damage than in normal aging. However, this remains a speculation until more research has been carried out in humans.

### Speech in noise

In the speech-in-noise test some subjects regarded 85 dB SPL listening level too loud and were measured at the lower level on both ears. Three subjects found even the lower listening level too loud and did not complete any speech test at all. The resulting number of measurements on the left ear was 125 at 70 dB SPL and 83 at 85 dB SPL, and on the right ear 108 and 72, respectively. ANOVAs were performed separately for the left and right ears with the S/N threshold as the dependent variable, with profession group as a between subjects factor, and with sound level as a within subjects factor.

*Industry* and *Education* were, on the left ear, significantly worse (p<0.05, Tukey's HSD) than *Music*, the only group that was normal at both levels. *Industry* was also significantly worse than *Other* (p<0.05, Tukey's HSD). However, when age was introduced as a covariate all the significant group differences disappeared. (The change in S/N at threshold per decade was 1 dB.)

**Figure 5:**
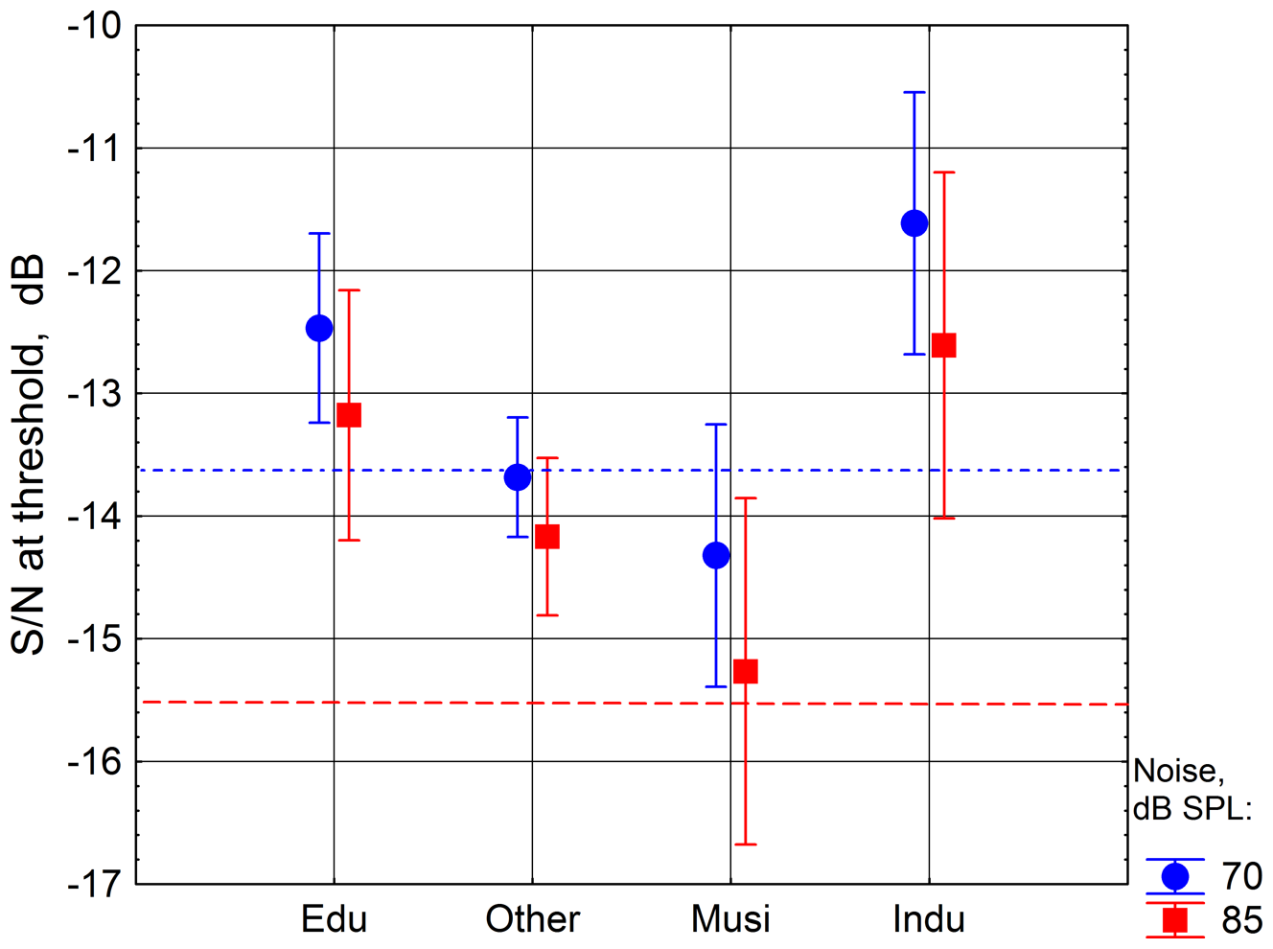
Mean of the speech recognition thresholds in noise. S/N at threshold for the profession groups, two noise levels 70 and 85 dB SPL, left ear. The vertical bars show 95% confidence intervals. Numbers of subjects in the different groups are from left to right 23, 58, 12, and 12. The horizontal lines show values for a *middle-aged reference group* without hearing problems for the noise levels 70 (dashed) and 85 (dotted) dB SPL. (The means and the numbers of measurements were obtained from the ANOVA, which explains the discrepancy from the number of measured ears.)

More important was that, still on the left ear, *Industry* and *Education* were markedly worse than the *middle-aged reference group* (p<0.0001 for both groups at 85 dB SPL, t-tests), see Figure 5, like in the forward masking results. The group *Other* was normal at 70, but not at 85 dB SPL. Note that both the *middle-aged reference group* and the *group of young women* had considerably better results on the speech-in-noise test at the presentation level 85 dB SPL than at 70 dB SPL. Also the test groups in the current study had better results at the higher level, but the improvement was smaller due to larger deteriorations at the higher level than at the lower level.

On the right ear all group means except *Industry*'s were within normal limits compared to the mean over right and left ear of our *middle-aged reference group* without hearing problems. However, only two subjects in *Industry* were tested on the right ear.

### Tinnitus matching

**Figure 6:**
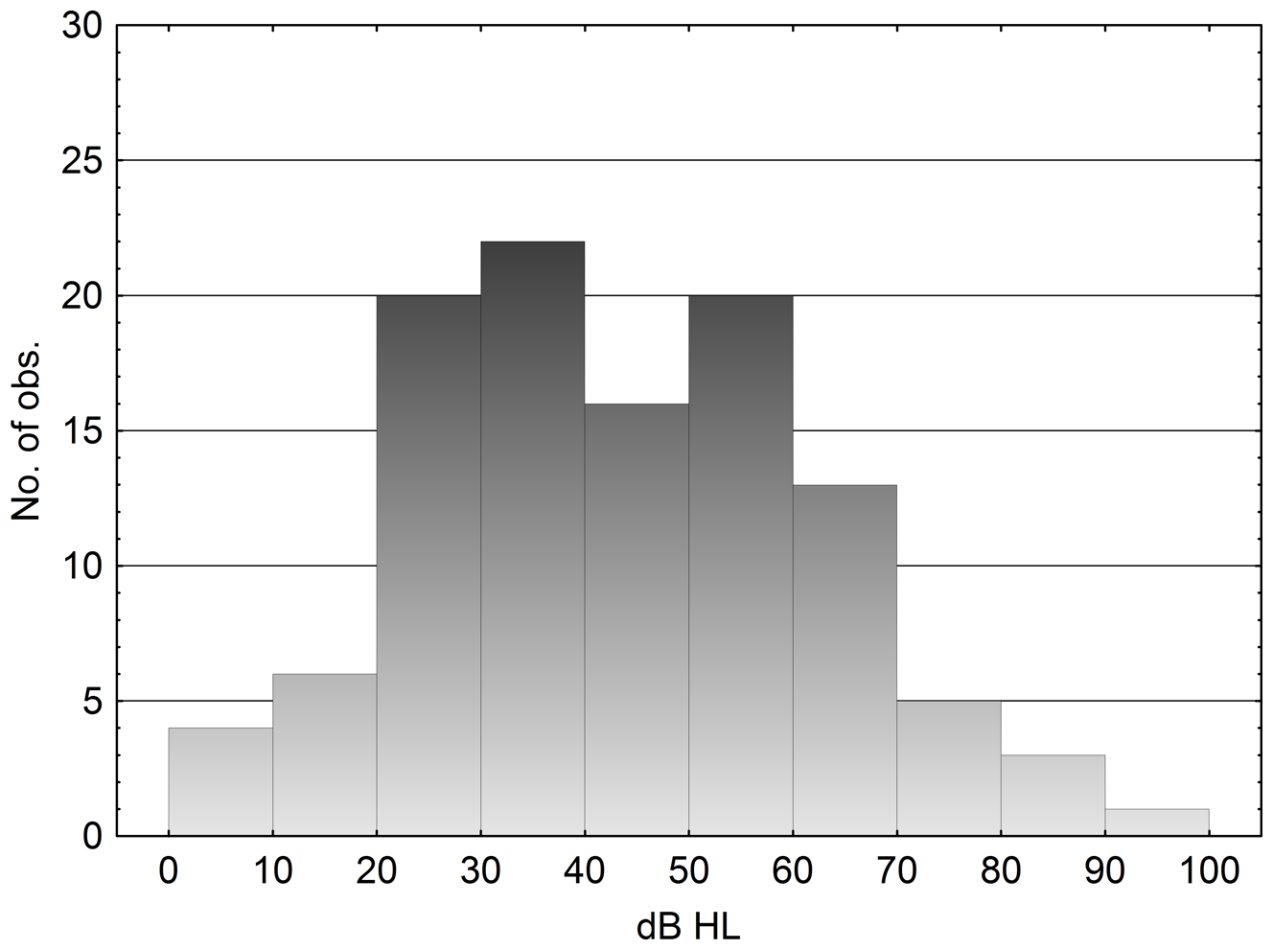
Histogram of matched tinnitus level, left ear, n = 110. Right ear's histogram (n = 89) was similar. Median levels for left and right ears were 40 and 38 dB HL, respectively.

An ANOVA was performed with the matched tinnitus level as the dependent variable, with profession group as a between subjects factor and with ear as a within subjects factor. The group *Industry* had significantly louder tinnitus than *Music* and *Other* (p<0.05, Tukey's HSD) according to the matching, but not significantly louder than *Education*. Figure 6 shows a histogram of the matched tinnitus levels for the left ear (n = 110). The results of the right ear were similar, as many subjects located their tinnitus in the centre of the head or in both ears. The subjects who did no matching to the test tone (n = 83) either had no pitch in their tinnitus or, for most of them; they had no tinnitus to match to at the test moment.

### Relations between measurements

Relations between measurements were tested with linear regression. When forward masking was involved it was used as the independent variable. There were significant negative, and similar, correlations between *forward masking* results at each of the three test levels and *matched tinnitus level* for both ears (at best r = −0.36, p<0.001 at 65 dB SPL, right ear). This means that louder tinnitus was correlated to poor forward masking. The two groups with louder tinnitus, *Industry* and *Education*, had significantly worse forward masking results than *Music* and *Other*. That subjects with worse forward masking had louder matched tinnitus is in concordance with reports of increased gain at higher levels in the auditory system following loss of IHCs[32]. This is also in accordance to the assumption that there is an increased risk of tinnitus and abnormal sensitivity to loud sound in case of neural degeneration proximal to intact IHCs[27], [28].

There was a significant correlation between the threshold difference in *forward masking* and the *speech recognition threshold in noise*. The groups with the poorest forward masking results (possibly also with the worst IHC status) had the worst speech recognition in noise. The correlation was strongest for both speech-in-noise levels on the right ear, and for the lower noise level on the left ear with correlations between −0.5 and −0.6 (p<0.00001) for relevant level combinations of speech-in-noise and forward masking results (with forward masking level equal to or lower than the noise level in the speech test). For the left ear at the higher speech-in-noise level the correlation was −0.3 to −0.4 (p<0.005).

Unlike the difference between the groups in the forward masking test, the differences between the groups in the speech-in-noise test were not significant when compensated for age. One may speculate that forward masking may be a basic measure of the status of receptor hair cells and a neural degeneration beyond, which has been shown to be a long-term after-effect of noise exposure[27], [28], [30], whereas speech recognition also has an added age-dependent component of cognition.

There were strongly significant correlations between *forward masking* results, only measured at 2000 Hz, and *PMTF*-results at the same frequency, indicating characteristics of the PMTF-curves concordant with hyper-PMTF (P-type = 3) or suspected hyper-PMTF (P-type = 2): Small forward masking threshold differences were accompanied by high maximum valley thresholds (r = −0.44, p<0.0001 for both ears and forward masking at 65 dB), and by positioning of the maximum valley thresholds at low noise level (r = 0.31, p<0.005 for both ears and forward masking at 65 dB SPL). The bond between forward masking and PMTF regarding incidents with sudden loud sound was further strengthened by the fact that high maximum valley thresholds were accompanied by positioning of the maximum valley thresholds at low noise level (p<0.00001, r = −0.49 for the left ear, r = −0.54 for the right ear), both typical for the hyper-PMTF, Figure 2D. There were also significant correlations between the forward masking variables and the corresponding PMTF variables at 4 kHz on the left ear.

### Relations between results of questionnaire and hearing tests

*Forward masking* proved to be the hearing test that showed the most revealing differences (or similarities) between persons with different professions. Therefore it was considered suitable to investigate if non-auditory factors could influence the forward masking results. Thus ANOVAs were performed with the forward masking threshold difference (difference between the thresholds of the brief tone at 5 ms and at 50 ms after the noise burst) as the dependent variable, and with ear and one non-auditory factor at a time as within subjects factors. The non-auditory factors were self-reported medication, headaches, neck problems, smoking or stress. The only non-auditory factor with a possible influence on forward masking was *medication* (p<0.05, p = 0.05, Kruskal-Wallis). The dose response indicates that this is not a spurious finding: The mean threshold difference for individuals taking more than 50 pain relieving tablets/year was 22 dB. For those taking 1–50 tablets/year the mean was 28 dB, and for those taking 0 tablets/year it was 29 dB. However, we have no explanation to this correlation between medication and forward masking. The fact that the *Education* group had forward masking results similar to the *Industry* group and not to the less noise exposed groups *Music* or *Other* could not be explained by this correlation, since there was no difference whatsoever in use of medication between any of the groups.

*Hypersensitivity to loud sound* as reported in the questionnaire (no or yes response) was related to *speech recognition in noise* at the lower test level, 70 dB SPL, right ear (p<0.005, simple ANOVA). Persons, who were hypersensitive to loud sound, had a better S/N, −14.1 dB, than persons who were not so sensitive, S/N = −12.4 dB. This effect may be caused by healthy OHCs combined with ipsilateral and contralateral regulatory systems with defective restraining capacity, resulting in higher levels in the cochlea [4], [33], [34]. OAE data showed such tendencies, but no significances. The hypothesis that persons hypersensitive to loud sound may have worse results on speech recognition in the 85 dB SPL noise than those not so sensitive could not really be tested, since there were too few persons tested at that level. Neither could any differences between the two categories in the amount of improvement with noise level be reliably analysed.

### Complementary discussion

The hearing tests in this study show, that individuals with hearing problems in the profession group *Other,* consisting mainly of medical staff and students, have small deviations from results normal for their age, and that the group *Industry*, used for comparison, has substantial deviations in all tests. This might have been suspected from the self-estimated noise exposure including profession and other risk factors.

In our previous study musicians had some dysfunctions associated to damage to OHCs and the regulatory system, and a few of them showed the typical signs of incidents with sudden loud noise[4]. In the present study the group *Music* showed the same indications, but in addition the results were very normal for the two new measurements, forward masking and speech recognition in noise. The forward masking results were even as good as the results of the (much younger) *group of young women*. Thus the *Music*-group, except a few individuals exposed to incidents, had good results regarding measurements associated to IHC-function. Musicians are acknowledged to work at sound levels at which there is a risk of NIHL according to the international standardisation[8], and our estimations of noise exposures suggested that the musicians should have more dysfunctional hearing than the teachers. However, the participating musicians seemed to have come from fairly controlled soundscapes without unexpected loud sounds from electronic systems. The changes in sound level in the music are expected by the musicians.

In contrast the *Education* group showed results similar to those of the *Industry* group, and worse than normal for middle-aged persons, regarding forward masking, and speech recognition in noise. The self-reported tinnitus level was about the same and worse than for the other two groups. There were also PMTF-results that may be associated to exposure to sudden loud sounds. According to animal research, one may speculate that there are lesions in the inner hair cell area as referred to in the result/discussion section. Although there is an uncertainty of the location of the possible lesions, this study has clearly shown that our *Education* group had poor temporal processing. Regarding the location of possible lesions in the hair cell areas and the regulatory system we have based the discussions on the literature. Unfortunately there is hitherto less literature regarding inner hair cells than outer hair cells, because of physiological difficulties to measure. Our findings seem to justify more studies in this area.

Our assumption was that the participants of the *Education* group had been exposed to lower noise levels than the *Industry* and *Music* groups. In the questionnaire, the estimates of self-assessed noise exposure supported that assumption. However, recent results from noise measurements in preschools show that preschool teachers are exposed to considerable noise levels[6]. In the seventeen preschools in that study the mean equivalent sound level over a working day was less than 80 dB(A), but the maximum was 85 dB(A), The mean rating of the noise was between “somewhat troublesome” and “very troublesome”. About 80% of the 101 teachers judged unexpected sudden changes of the sound level to occur “several times per day” to “several times per hour”. It is reasonable that noise levels and noise characteristics, e.g. the content of sudden, unexpected loud noises, depend on type of school, age of pupils, classroom acoustics and cultural factors. The teachers in our study had not used hearing protection, although it may be used by for example woodwork teachers.

It is possible that many of the teachers may have come from soundscapes like the one described above, with noise described as troublesome but still tolerable, with several unexpected sudden changes of the sound level every working day. Other reasons for the *Education* group having worse results than the *Music* group could not be found in the analyses of answers to the questionnaire. Self-reported neck problems, pain relieving medication, eye and hair colour, smoking, and relatives with hearing problems were about the same in the two groups. The self-reported amounts of stress and tension headaches were slightly higher for these two groups than for the other groups.

In the study of risk factors for tinnitus in a population 55 years and older a number of extrinsic and health factors that could be linked to tinnitus were described[35]. The largest single factor was reported occupational tolerable noise exposure (9.3%), which may correspond to what Sjödin reported for preschools[6]. The conclusion was that a number of occupations with less noise burden than industrial exposure are at risk for noise-induced tinnitus. Another study found poor temporal processing and speech recognition in adverse listening conditions in individuals exposed to occupational noise more than 80 dB(A), but still with pure tone thresholds less than 25 dB HTL[36]. Those skills deteriorated with age, but were worse for exposed groups than for unexposed controls. Also that study seems to have relevance for the tested groups of teachers.

Our results show that persons exposed to moderate noise levels at work may run the risk of hearing dysfunction. Mean results of the 48 teachers in two measurements as well as of individual musicians and hospital staff with moderate noise exposure seem to prove that.

This study may have found dysfunctions that are characteristic for these groups before the audiogram is affected, but it will not give us a general overview of the effects of noise on hearing for these groups of professions at later stages of deterioration of the audiogram. Neither can it tell how common the dysfunctions are.

## Conclusions

In this study, individuals having auditory problems and normal or near-normal hearing thresholds were divided into groups of subjects with similar noise exposure, and measured with advanced hearing tests. For each type of measurement there were individuals with abnormal results possibly indicating cochlear dysfunction.

For many of the subjects these dysfunctions caused less improvement than normal when the listening level in the speech in noise test was increased. Subjects sensitive to loud noise had significantly better speech recognition in noise at the lower test level than subjects not sensitive.

There were characteristic results:

Teachers had results suggesting substantial dysfunction in the auditory system reflected in far worse forward masking and speech recognition in noise than a group of middle-aged without hearing problems. These results, suggesting poor temporal processing, were about equally poor as those of a group exposed to industrial noise. The latter group was tested for comparison and had about 20 dB worse pure tone thresholds. The matched tinnitus level was correlated to the forward masking results and those two groups had the loudest matched tinnitus, possibly caused by dysfunction in the inner hair cell area.

Musicians showed some deficits normally associated to outer hair cells and had good results for their age at forward masking, and so did a group mainly consisting of hospital staff and students. The musicians had normal speech recognition in noise at both listening levels.

The study suggests that persons exposed to occupational noise below or around risk levels may risk hearing dysfunction. Several of the teachers in this study are examples of that, possibly because of combinations of unfavourable working environment and individual susceptibility. Medication or other self-reported non-auditory factors could not explain the poor results of the teachers.

## Supporting Information

Appendix S1 **Questionnaire.** English version.
(DOC)

Appendix S2 **Estimation of noise exposure.**
(DOCX)
