# Supplementary material for: Tinnitus and Other Auditory Problems – Occupational Noise Exposure below Risk Limits May Cause Inner Ear Dysfunction
Source: PLoS One. 2014 May 14;9(5):e97377. doi: 10.1371/journal.pone.0097377 (PMC4020865; doi:10.1371/journal.pone.0097377)
Supplement: Appendix S1 — Questionnaire. English version. (DOC) [file pone.0097377.s001.doc]

Date: …………………….

Age: …........ years of age.

Occupation:..............................………..

*Figures are number of responses from the group of 193 subjects.
Text in italics is information related to the analyses.*

**QUESTIONS ABOUT HEARING**

**1. Have you experienced hearing loss ?** NO  72

YES  118

right ear  84 left ear  83 *(*66 *of them had ticked both ears.)*

always  30

often  26

occasionally  42

only after loud sounds  13

If so, under which circumstances ? .............................................................................

How long did it last?...................................................................

**2. Have you had ear infections?** NO ****  YES 

**Any other ear disease?** NO **** YES 

*Yes on one or both questions* 98. *No on both questions* 86.

**3. Do you experience tinnitus (tonal/noise) ?** NO  16

YES **** 176

centered in my head **** 93 in right ear only  20 in left ear only  25, *R+L* 33

always  120

often  39 how often ? ...........................................

occasionally  12 for how long? .......................................

only after loud sounds  5 for how long? .......................................

Describe the ***character*** of your tinnitus (and if possible, the pitch):: .........................................................................................................................................................

.........................................................................................................................................................

.........................................................................................................................................................

Do you find your tinnitus annoying? NO  11

YES  165

slightly annoying  63 rather annoying  47 very annoying  54

**4. Do you consider yourself hypersensitive to loud sounds?** NO  45

YES  146

right ear  103 left ear  106 *(*90 *of them had ticked both ears.)*

always  35

often  57

occasionally  49

only after loud sounds  4

**5. Have you experienced distortion of sound ?** NO  144

YES  46

right ear  28 left ear  33 *(*20 *of them had ticked both ears.)*

often  8 occasionally  33

Please describe situations!

.......................................................................................................................................................

....................................................................................................................................................

**6. Are you affected/annoyed by your hearing problems?** NO  13

YES **** 174

only in quiet  49 in ordinary environments, but masked by stronger sounds  60

always, disturbs sleep, affects my whole life  52 Other:
......................................................................................................................................................
......................................................................................................................................................

**7a. Are you exposed to loud noise at work?** NO  106

YES  83

Please describe!
......................................................................................................................................................
......................................................................................................................................................

**7b. Are you exposed to loud noise in leisure time?** NO  124 YES  68

Please describe.....................................................................................................................................
......................................................................................................................................................

**8. Musical activities:** I’m playing myself NO  121 YES  70
what ?:…………………………………………………………………………………..

I go to concerts/disco NO  79 YES  102

listen to walkman/car stereo NO  55 YES  126

**9. Have you done military service?** NO  127 YES  63 Please describe!......................................................................................................................................

**10.** **Have you had an accident with loud impulse noise, at work or in leisure time,
e.g. banger/shot near the ear or a box on the ear, that gave a sense of fullness and/or tinnitus?**

NO  82 YES  108 Please describe!

......................................................................................................................................................
......................................................................................................................................................

**11. Do you use pain killers** (e.g. Treo, Magnecyl, Aspirin)?
Less than 50 a year  104 More than 50 a year  24 Never  64

**12. Do you take medication for tinnitus** NO  180 YES  10

**if yes: what ?** *Various. (5 subjects in group Other, 3 in Music, 1 in Education , 1 in Indu)*

**Have you taken that medication today ?** NO  YES  *not analysed*

**13. Do you have problems with bite or jaws?** NO  108 YES  82 Please describe!

....................................................................................................................................................

**14. What colour is your hair (or was, before becoming grey, bald or dyeing it) ?**

Red  Blond  Dark  Black 

*Many have ticked two adjacent boxes! Coded as degree of pigmentation 1, 1.5, 2, 2.5, 3, 3.5, 4*

*with resp. number of answers* 4, 2, 86, 17, 72, 3, 8

**15. Which is your eye colour ?** Blue  Brown  Green  Grey 

*Many have ticked two adjacent boxes! Coded as degree of pigmentation 1, 1.5, 2, 2.5, 3, 3.5, 4*

*with resp. number of answers* 34, 6, 33, 7, 15, 3, 94

**16. How is your skin affected by sunbathing ?**  Red/burnt, tanning slowly or not at all 28

 Slightly red/burnt, tanning normally 140

 Never red/burnt, get a darker tan than most people 23

**17. Smoking:**

**a. Smoking habits ? (Note! More than one alternative possible)**

 I have never smoked 97  I smoke 70  I smoked before 19

 I started to smoke in (year)**………….**  I stopped smoking in (year) **……………**

**b. If you do/did smoke, how much
 did you smoke ?**  No. of cigarettes/day **………..**

 No. of grams of pipe tobacco/week **……….**

 No. of cigars/day **………….**

 No. of cigarillos/day **………...**

*Not analysed.*

18. Have you experienced scull injury
 and/or fracture of the scull  No 143  Yes 37  Vet ej 12

**19. Do you have
 neck problems/ache ** No 95 **** Yes 97

**tension headache** **** No 116 **** Yes 74 **or been subjected to a whiplash injury ? ** No 162 **** Yes 19

**20. Do you feel very stressed/anxious?
 ** Never 8 **** Seldom 42 **** Sometimes 71 **** Often 61 **** Always 10

**21. Do/did you have relatives with early hearing loss or tinnitus ?
** No 57 **** Yes 81 **** I don’t know 64

**………………………………………………………………………………………………………….**

**22. Is Swedish your mother tongue?** Yes  169 No  21

**if No, which: ………………………………………………**
