# Supplementary material for: Tinnitus and Other Auditory Problems – Occupational Noise Exposure below Risk Limits May Cause Inner Ear Dysfunction
Source: PLoS One. 2014 May 14;9(5):e97377. doi: 10.1371/journal.pone.0097377 (PMC4020865; doi:10.1371/journal.pone.0097377)
Supplement: Appendix S2 — Estimation of noise exposure. (DOCX) [file pone.0097377.s002.docx]

Estimation of noise exposure

The total noise exposure was calculated as the sum of the three figures for Occupational noise, Leisure noise and Compulsory military service, which gave a scale from 0 to 9.
Self-reported incidents with impulse noise were treated separately.

Item numbers refer to the questionnaire.

# Occupational noise exposure

Item #7a

## Industrial sector

Grading: 0 - 4

2 - 3: part time; short professional experience; combinations of administrative and industrial work

4: combinations of severe exposure to continuous nosie and impulse noise

E.g.: mechanics; sheet metal workers; construction,

## Transporation *(Not enough subjects within threshold fence)*

Grading: 0 - 4

4: helicopter pilots

## Service sector *(Not enough subjects within threshold fence)*

Grading: 0 - 3

Restaurants; school kitchens,

## Educational sector

Grading: 0 - 3

0 - 1: Primary shool teachers; high scool teachers (theoretical subjects); principals

1 - 2: Pre-school teachers; PE (physical excercise) teachers,

2 - 3: Wood- and metalwork teachers; vocational teachers

## Music sector

Grading: 0 - 3

1 - 2: part time musicians; short professional experience (<20 years)

2 - 3: full time musicians, sound technicians, music teachers, long professional

experience (>20 years)

***Noise exposure of subjects not working in the listed sectors was judged according to the same principles. Please note that some of the sectors above are not represented in the final test groups. There were not enough subjects in some sections with good enough hearing thresholds.***

# Leisure time noise exposure

Items #7b, 8. Grading: 0 - 3

0: attending concerts, discoes; listening to individual devices only; both exposures: 1

1: Play music non-professionally; excessive listening to music

2 - 3: shooting, hunting; motor sports; combinations

# Compulsory military service

Item #9. 0 - 2

No: 0

Yes: 1: ordinary service; 2: exposed service

Persons with only marginal noise exposure in compulsory military service, being well protected during a few initial shooting exercises, got the estimate 0, the same as persons not having been in military service.

# Acute noise trauma exposure

Item #10. Grading: 1 - 4

3 - 4: severe problems afterwards; repeated exposures; includes some occupations in item #7a, mechanics, metal sheet workers.
